# VEGF sticky-trap: the first report of a non-systemically acting angiogenesis inhibitor

**DOI:** 10.1002/emmm.201404026

**Published:** 2014-05-06

**Authors:** David M Favara, Adrian L Harris

**Affiliations:** Molecular Oncology Laboratories, Weatherall Institute of Molecular Medicine, University of OxfordOxford, UK

## Abstract

Current therapeutic anti-angiogenic biologics used for the treatment of pathological ocular angiogenesis such as in diabetic retinopathy and wet macular degeneration often lead to detrimental side effects due to their interference with normal blood vessel physiology. In this issue of *EMBO Molecular Medicine*, Michael *et al* report on a novel angiogenesis inhibitor with unique properties that allow for local inhibition of angiogenesis without detectable systemic side effects.

Pathological angiogenesis occurs in a wide spectrum of disease, ranging from cancer to inflammatory disorders (Potente *et al*, 2011). Within the eye, abnormal retinal angiogenesis commonly occurs in diabetic retinopathy (DR), wet macular degeneration (WMD) and in newborn infants with retinopathy of prematurity (ROP). These ocular conditions all manifest excessive production of the pro-angiogenic growth factor VEGF-A, which if left untreated results in permanent blindness. In these conditions, inhibiting VEGF has been shown to improve clinical outcome (Chen *et al*, 2011; American Diabetes Association, 2013; Smith & Kaiser, 2014).

Current approaches (Cook & Figg, 2010) to inhibiting VEGF centre around (1) neutralizing monoclonal antibodies against VEGF; (2) small multi-kinase inhibitors; and (3) soluble VEGF decoy receptors such as recombinant VEGF-trap. Although inhibition of angiogenesis in cancer needs to be systemic because of multiple metastatic sites, in certain non-malignant cases, inhibition of angiogenesis needs to be localized to a specific organ or tissue. The systemic effects of the above approaches often lead to detrimental side effects, exacerbated in elderly patients with multiple co-morbidities, pregnant women and patients undergoing surgery. Premature neonates, having prematurity-associated retinal VEGF excess, still require physiological VEGF levels to further pulmonary and renal maturation between 30 and 40 weeks of postmenstrual age. The search and need for novel, locally acting anti-angiogenic therapies is thus great.

systemic effects often lead to detrimental side effects

In this issue of *EMBO Molecular Medicine*, Michael *et al* (2014) present the first report of a novel locally acting angiogenesis inhibitor. This new inhibitor, named VEGF Sticky-trap, has been designed to take advantage of the strong interactions that occur between the heparin-binding domains (HBDs) found on VEGF exon 6 & 7 and the heparan sulphate proteoglycans (HSPGs) found in the extracellular matrix (ECM). By genetically fusing the original VEGF-trap (Holash *et al*, 2002) (with modifications to decrease serum half life) to VEGF's HBDs, the authors have engineered a novel VEGF-trap that exerts local anti-angiogenic activity through its ability to bind (stick) to the ECM's HSPGs at the site of its administration. This ensures that following local administration into an organ or tissue, VEGF Sticky-trap remains at the site of delivery and does not leak into the circulation.

VEGF Sticky-trap comprises 3 components (Fig 1): (1) A VEGF-trap region containing the IgG-like regions from VEGF-R1 and VEGF-R2 as per the original VEGF-trap; (2) a modified Fc region where the CH2 domain has been removed to ensure shorter serum half life (replaced with a hinge domain and poly-glycine-serine linker; and (3) the ‘sticky’ region, which contains HBDs from VEGF's exons 6 & 7 alongside exon 8 (necessary for correct disulphide bond formation of the HBD).

**Figure 1:**
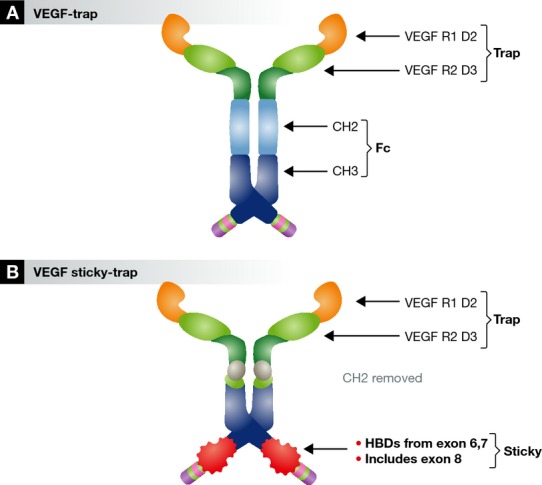
(A) The structure of the original VEGF-trap (Holash *et al*, 2002). (B) The structure of VEGF Sticky-trap (Michael *et al*, 2014). VEGF, vascular endothelial growth factor; VEGR R1 D2, VEGF receptor 1 domain 2; VEGF R2 D3, VEGF receptor 2 domain 3; CH, constant heavy domain; HBD, heparin-binding domain. Illustration modified from reference (Michael *et al*, 2014).

Michael *et al* elegantly present VEGF Sticky-trap as a novel, effective and safe therapeutic for DR and ROP. To do this, they systematically assess VEGF Sticky-trap's function, ability to remain localized, effect on angiogenesis, systemic toxicity, and its localization and effect in the mouse models of DR and ROP. Experiments were conducted with VEGF Sticky-trap, VEGF-trap, VEGF-trap variants and appropriate negative controls.

Through the use of systemic pharmacology, the authors show that following subcutaneous administration in mice, VEGF Sticky-trap is rapidly metabolized, remains undetectable in the circulation and does not cause a circulating VEGF spike (in contrast to VEGF-trap). Post-mortem examination 48 h following administration revealed no detectable VEGF Sticky-trap deposits in a range of tissues and organs. In addition, VEGF Sticky-trap is shown to be a greater inhibitor of tumour microvascular density than traditional VEGF-trap when expressed in cancer cell line xenografts in nude mice, further strengthening the argument that VEGF Sticky-trap is tightly retained and metabolized at its site of administration.

Systemic toxicity profile experiments reveal VEGF Sticky-trap to have no effect on either wound healing, systemic vascular biology or glomerular integrity, producing the same results as negative control non-tumour-bearing mice. This contrasts to VEGF-trap, which impairs wound healing, causes a fivefold increase in empty-sleeve formation in the tracheal microvessel immunostaining assay and produces decreased glomerular perfusion and implosion following high subcutaneous dosage. VEGF Sticky-trap's enhanced safety profile is most probably also due to its localized retention.

With regard to the eye, the authors reveal that VEGF Sticky-trap is more effective than VEGF-trap in inhibiting pathological angiogenesis. This was assessed using the oxygen-induced retinopathy (OIR) mouse model for DR and ROP, revealing lower vaso-obliteration and tuft formation in the eyes treated with intra-vitreal VEGF Sticky-trap compared to VEGF-trap. Additionally, of all the traps tested, only VEGF Sticky-trap was detectable in the eye 48 h following administration. Interestingly, VEGF Sticky-trap remains in the eye for up to 12 days following intra-vitreal injection, longer than any other agent used to inhibit ocular angiogenesis. This is particularly exciting as it may allow for wider dosing intervals. A pre-clinical trial investigating the efficacy of various VEGF Sticky-trap dosing intervals compared to conventional ocular VEGF inhibitor therapy will be needed to confirm this.

Furthermore, the authors note that intra-vitreally administered VEGF Sticky-trap localizes to the inner-limiting membrane, lens and ciliary body. This is of clinical importance as the inner-limiting membrane is the site of abnormal angiogenesis in both ROP and DR. With subretinal administration, VEGF Sticky-trap remains localized in the subretinal space, the site of abnormal angiogenesis in WMD. Lastly, the authors reveal that VEGF sticky-trap is not toxic to the eye and does not interfere with photoreceptor function or retinal and choriocapillaris integrity. This was assessed using electroretinography and electron microscopy following three intra-vitreal injections of VEGF Sticky-trap into one eye and then compared to the non-exposed contralateral eye.

A possible limitation is whether VEGF Sticky-trap would distribute to all the required areas in the human eye, a much larger structure than any investigated in this study. Additionally, it may be difficult to control dosing because of variable retention in different patients at different stages of disease, bearing in mind that only inbred animals have been studied so far. However, these issues can only be investigated in the clinical context.

VEGF Sticky-trap thus represents a new and unique, locally acting VEGF-inhibitory molecule with exceptional pharmacological, safety and efficacy properties

Taken all together, VEGF Sticky-trap thus represents a new and unique, locally acting VEGF-inhibitory molecule with exceptional pharmacological, safety and efficacy properties. VEGF Sticky-trap might well meet a major need for locally acting ROP, DR and WMD therapy and is likely to be a significant step forward in anti-angiogenic therapy. We believe that VEGF Sticky-trap holds great promise as a strategy that could be rapidly translated into clinical practice. Additionally, we expect that VEGF Sticky-trap (and future related molecules) will have significant impact on the field of tumour biology in local control of recurrent disease. The future appears very bright for this novel construct.

## References

[b1] American Diabetes Association (2013). Standards of medical care in diabetes-2013. Diabetes Care.

[b2] Chen J, Stahl A, Hellstrom A, Smith LE (2011). Current update on retinopathy of prematurity: screening and treatment. Curr Opin Pediatr.

[b3] Cook KM, Figg WD (2010). Angiogenesis inhibitors: current strategies and future prospects. CA Cancer J Clin.

[b4] Holash J, Davis S, Papadopoulos N, Croll SD, Ho L, Russell M, Boland P, Leidich R, Hylton D, Burova E (2002). VEGF-Trap: a VEGF blocker with potent antitumor effects. Proc Natl Acad Sci U S A.

[b5] Michael IP, Westenskow PD, Hacibekiroglu S, Cohen Greenwald A, Ballios BG, Kurihara T, Li Z, Warren CM, Zhang P, Aguilar E (2014). Local acting Sticky-trap inhibits VEGF dependent pathological angiogenesis in the eye. EMBO Mol Med.

[b6] Potente M, Gerhardt H, Carmeliet P (2011). Basic and therapeutic aspects of angiogenesis. Cell.

[b7] Smith AG, Kaiser PK (2014). Emerging treatments for wet age-related macular degeneration. Expert Opin Emerg Drugs.

